# Quality of Life, Mental Health, and Illness Perception in Pediatric Food Allergy

**DOI:** 10.3390/children12121657

**Published:** 2025-12-06

**Authors:** Laura Polloni, Lucia Ronconi, Sabrina Bonichini, Irene Degola, Roberta Bonaguro, Francesca Lazzarotto, Alice Toniolo, Beatrice Serra, Rossana Schiavo, Antonella Muraro

**Affiliations:** 1 Food Allergy Referral Centre, Veneto Region, Department of Women’s and Children’s Health, Padua University Hospital, 35128 Padua, Italy; roberta.bonaguro@aopd.veneto.it (R.B.); francesca.lazzarotto@aopd.veneto.it (F.L.); alice.toniolo@aopd.veneto.it (A.T.); beatrice.serra1@edu.unifi.it (B.S.); muraro@centroallergiealimentari.eu (A.M.); 2 Unit of Psychology, Padua University Hospital, 35128 Padua, Italy; rossana.schiavo@aopd.veneto.it; 3 Computer and Statistical Services, Multifunctional Pole of Psychology, University of Padua, 35131 Padua, Italy; l.ronconi@unipd.it; 4 Department of Developmental and Socialization Psychology, University of Padua, 35131 Padua, Italy; s.bonichini@unipd.it; 5 School of Psychology, University of Padua, 35131 Padua, Italy; irene.degola@studenti.unipd.it

**Keywords:** food allergy, anaphylaxis, children, parents, quality of life, illness perception, psychosocial burden, mental health, emotions, anxiety, psychological wellbeing

## Abstract

**Highlights:**

**What are the main findings?**

- Among children with food allergy (FA), quality of life (QoL) burden is positively associated with mental health difficulties, parental illness perception, and more allergens. Previous anaphylaxis is associated with a higher FA identity.
- Higher internalizing problems, emotional representation of the illness, and timeline are associated with a poorer QoL for the child.

**What are the implications of the main findings?**

- The results highlighted the importance of understanding and exploring illness perception as well as paying attention to the psychosocial–emotional aspects of FA in both children and parents.
- Clinicians should investigate illness perceptions of caregivers and offer evidence-based information, discussion of doubts and concerns, training in FA and anaphylaxis management, and, when appropriate, referral for psychological support.

**Abstract:**

**Background/Objectives:** The beliefs about a disease and its treatment determine how patients and caregivers manage and adapt to the illness. The study aimed to explore the QoL and mental health of children with food allergy (FA), and parental illness perception, analyzing influences of sociodemographic and clinical factors and associations between constructs. **Methods:** This cross-sectional study involved 79 parents of children (3–12 years) with FA, who completed the Food Allergy Quality of Life Questionnaire—Parent Form (FAQLQ_PF), Strengths and Difficulties Questionnaire (SDQ), and Brief Illness Perception Questionnaire (B-IPQ). Pearson correlation coefficient and multiple linear regressions were performed. **Results:** FAQLQ score was positively associated (0.28) with SDQ score, particularly internalizing problems (0.33), and with B-IPQ score (0.64), consequences for the child and parents (0.66), timeline (0.43), and emotional representation (0.63). SDQ score was negatively associated with parental control (−0.27) and coherence (−0.24), while internalizing problems were negatively associated with parental control (−0.23) and positively associated with timeline (0.24). A greater number of allergens was associated with a worse QoL (*p* < 0.05). Previous anaphylaxis was associated with higher illness identity (*p* < 0.05). An age between 7 and 12 years was associated with lower control and coherence. In the final model, higher scores on internalizing problems, timeline, and emotional representation were associated with poorer child QoL (*p* < 0.001). **Conclusions:** It is crucial to understand and explore illness perception, as well as focus on psychosocial–emotional aspects of FA in both children and parents. A multidisciplinary approach addressing medical and psychological aspects of FA should be implemented to ensure optimal QoL.

## 1. Introduction

Food allergy (FA) has been defined as adverse reactions to food in which immunologic mechanisms have been demonstrated, and it can result in considerable morbidity, including life-threatening anaphylaxis [1]. FA is a major public health problem, affecting approximately 8% of children in the United States. This percentage rises to 11% when considering FA reported by parents, without confirmation by a specialist [2,3]. Indeed, studies highlight a large discrepancy between medically diagnosed FA and reports of FA symptoms by care seekers [4,5]. Across Europe, a significant percentage, ranging from 11% to 28.7%, of children aged 7 to 10 years exhibit IgE sensitization [6]. After diagnosis, patients and families must address allergen avoidance, making appropriate substitutions, be alert to possible contamination, recognize allergic symptoms, and always carry medications, including self-injectable adrenaline [7]. This can impact the daily routines, social lives, and emotions of patients and caregivers. Over the past two decades, research has increasingly focused on understanding how FA affects the quality of life (QoL) and psychosocial wellbeing of children and adolescents [8,9]. The results showed that pediatric patients with FA not only reported a lower QoL but also, in some cases, increased mental health symptoms [8,10,11,12,13,14,15,16,17]. The burden experienced by children and adolescents with FA appears to largely stem from the social consequences of the condition and concerns about accidental exposure [8,10,17,18]. It has also been found that older children and those with more severe FA report a greater negative impact [8,9,17]. Furthermore, the parental perceptions about the illness of a child may have a significant impact on the QoL of the whole family. Studies on chronic illness have demonstrated that parents develop cognitive and emotional representations, which then lead to the adoption of specific coping strategies [19,20]. The beliefs of the patients and their parents about a disease and its treatment determine how they manage and adapt to the new situation created by the diagnosis. Patients and their caregivers modify their beliefs, feelings and behaviours in ways that may affect the illness outcome, their compliance with treatment and their QoL [20]. Previous studies in chronic patients, including allergy sufferers, have found that greater perceived consequences, identity, emotional response, and concern about the illness are associated with poorer psychological, social, and physical functioning and worse disease outcomes [19,20,21,22]. The perception of strong personal control over the illness is associated with lower levels of stress. However, only one Chinese study [23] has measured illness perception in parents of children with FA, finding higher scores on the dimensions related to illness consequences, timeline, and concern. There is no data, to our knowledge, on the relationship between illness perception and psychosocial impact in families with FA. Understanding the factors that can influence the QoL allows us to improve the wellbeing of patients and their caregivers. Therefore, the present study aims to explore the QoL and mental health of children with IgE-mediated FA, assessed by the parents, as well as the parental perception of the disease, analyzing the possible influence of sociodemographic and clinical factors and the possible association between the constructs. Our hypothesis is that, as in other chronic diseases, the QoL of the child may be associated with psychological wellbeing, parental perception of the disease, and the severity of FA.

## 2. Materials and Methods

### 2.1. Study Design

The present study used a quantitative cross-sectional design, using validated questionnaire measures assessing QoL, child mental health, and parental illness perception about FA.

### 2.2. Participants and Procedure

The study involved 79 parents of pediatric patients (aged 3–12 years) with a medical diagnosis of immunoglobulin E (IgE)-mediated FA. Patients with concomitant non-allergic diseases and parents who did not speak Italian were excluded. Sociodemographic characteristics and clinical variables of pediatric patients and their parents are reported in Table 1.

Participants were recruited into the study during their children’s outpatient visits at the Food Allergy Referral Centre of the Veneto Region in Padua (North-Eastern Italy) sequentially over a 6-month period. Eligible parents were provided with an explanation of the study, its purposes, and procedures. If they wished to participate voluntarily, they were asked to sign the informed consent form and complete all sections of the questionnaires (missing data were not allowed). A researcher was available to answer any questions about the study and to verify that the survey was complete. Clinical data were collected through a double check between medical records and parental reports. A total of 102 eligible parents were invited to participate, and 79 (77.45%) accepted and provided written consent. The study was conducted in accordance with the Declaration of Helsinki. The research protocol was approved by the Psychological Research Ethics Committee of the University of Padua (Ethical approval code: 8F38BDEAF4972275113C1FE7100AD2FC; Approval date: 8 October 2015; Protocol number: 1707) as part of a larger project on quality of life and the management of food allergies and anaphylaxis in the family. All participants provided their written informed consent before enrolment. Personal data was managed in full compliance with privacy regulations in accordance with local legislation (Legislative Decree No. 196/2003).

### 2.3. Instruments

Parents completed the following validated measures:

• *The Food Allergy Quality of Life Questionnaire—Parent Form (FAQLQ-PF)* [24,25] is a validated and reliable disease-specific questionnaire that assesses the health-related quality of life (HRQoL) of children with FA aged 0–12 years old from the parents’ perspective. The FAQLQ-PF has been translated and validated in various languages worldwide, demonstrating excellent validity and reliability regarding patient HRQoL. The number of items on the questionnaire varies with age: 14 items (0–3 years), 25 items (4–6 years), or 30 items (7–12 years), with a response scale ranging from 0 (no impact on HRQoL) to 6 (maximal impact on HRQoL), with higher scores indicating more HRQL impairment. Items may be grouped into three subscales: general emotional impact, food-related anxiety, and social and dietary. The total score is calculated as the mean of the three subscales (range score 0–6).
• *The Strengths and Difficulties Questionnaire (SDQ)* [26,27] https://www.sdqinfo.org/py/sdqinfo/b3.py?language=Italian (accessed on 1 January 2019) is a validated and widely used instrument consisting of 25 items to assess emotional symptoms, behavioural problems, hyperactivity and inattention, peer problems, and prosocial behaviour in children and adolescents aged 4 to 16 [26]. Respondents indicate on a 3-point Likert scale (0–2) how much each symptom applies to their children. Higher scores denote greater problems except for the prosocial domain, for which a higher score indicates more positive behaviour. The Total Difficulties score ranges from 0 to 40 and is calculated by adding the scores obtained on the scales relating to emotional, behavioural, hyperactivity and peer problems. The individual scales, such as emotional symptoms or hyperactivity, score 0 to 10. It is also possible to calculate an externalizing score by adding up the scales relating to conduct and hyperactivity (range score: 0–20), and an internalizing score by adding up the scales relating to emotional and relational problems (range score: 0–20).
• *The Brief Illness Perception Questionnaire (B-IPQ)* [28,29,30] is a validated tool to assess cognitive and emotional representations of a disorder. It consists of nine dimensions:
  − Consequences: The expected effects of the illness on the individual’s life.
  − Timeline: The perceived duration of the illness.
  − Identity: The symptoms a person associates with the illness.
  − Personal Control: The belief that the individual can control their illness.
  − Treatment Control: The belief that the illness can be managed or cured through treatment.
  − Concern: The emotional response to the illness, such as worry or fear.
  − Emotions: The emotional impact of the illness.
  − Coherence: The degree to which a person understands their illness.
  − Cause: Personal ideas about the origins of the illness.

Responses are given on a 0–10 Likert scale. A higher total score indicates a stronger perception, with a possible score range of 0 to 80. The last item is an open-ended question that asks participants to report, in their opinion, possible causal factors of the illness. In agreement with the author of the questionnaire, the wording of the questions was adapted to assess parents’ perceptions of FA specifically.

### 2.4. Statistical Analysis

First, descriptive statistics were calculated for the main variables of this study—QoL, mental health of children with FA, and parental illness perception—and the association between these variables was investigated using the Pearson correlation coefficient. Next, the influence of sociodemographic and clinical factors on these main variables was explored using multiple linear regression. Finally, the additional contribution of child mental health and parental illness perception to child QoL was assessed, controlling for sociodemographic and clinical factors.

## 3. Results

### 3.1. Descriptives

Parents’ and children’s characteristics are shown in Table 1. The participating parents had a mean age of 42.94 (SD = 5.75; range 31–58 years), 53% were mothers, and most had a high school diploma (68%). Children with FA were mostly male (56%) with a mean age of 7.97 (SD = 2.85; range 3–12 years). Most (84%) were multiallergic, and 86% had had previous anaphylaxis. With regard to QoL concerns, as shown in Table 2, parents reported overall and subscale FAQLQ-PF scores for their children with FA ranging from 2.98 to 3.15 (on a scale from 0 = no impact to 6 = maximum impact). The total SDQ score for emotional–behavioural difficulties attributed by parents to their children had a mean value of 10.99 (SD = 5.88), which falls within the normal limits (0–13), as did the subscale scores. The two macrocategories of difficulties, internalizing and externalizing problems, were observed to be similar. The dimensions of the illness (B-IPQ scores) that parents perceived as having the greatest impact were consequences for the child and parent, timeline, identity, concern, and emotional representation. Conversely, those perceived as having the least impact were personal control for the child and parent, treatment control, and coherence. Finally, 49% of parents, in the open-ended question about the cause of the disease, indicated genetic factors over other factors (e.g., environmental causes, stress, and chance).

### 3.2. Correlations Between Psychological Measures (FAQLQ-PF, SDQ, and B-IPQ Scores)

As shown in Table 2, a strong positive correlation was found between the FAQLQ-PF subscales, as well as between the SDQ subscales and macrocategories of internalizing and externalizing problems. For this reason, we focused on the total scores of these instruments in examining the correlations and in the subsequent analyses. The QoL impairment (FAQLQ-PF total score) was moderately and positively associated with the child’s overall difficulties (SDQ total score), particularly internalizing problems. Furthermore, it was moderately and positively associated with the B-IPQ total score and some aspects of illness perception, such as consequences for the child and parents, timeline, and emotional representation. The child’s overall difficulties were negatively associated with two B-IPQ dimensions, personal control of parents and coherence, while internalizing problems were weakly negatively associated with personal control of parents and positively associated with timeline. No correlation was found with the indication of genetic factors as the cause of the disease (*p* > 0.05).

### 3.3. Influence of Sociodemographic and Clinical Factors on FAQLQ-PF, SDQ, and B-IPQ Scores

As reported in Table 3, only a small portion of the variance in children’s FAQLQ-PF total score and SDQ total score, between 7% and 13%, was attributed to sociodemographic and clinical factors. Only a greater number of allergens was moderately associated with a worse QoL in the child (Beta = 0.27; *p* = 0.024). Similarly, in some dimensions of illness perception, we found a low percentage of variance explained by sociodemographic and clinical factors, ranging from 8% to 14%. Specifically, male parenthood was moderately associated with a lower perception of parental consequences (Beta = −0.27; *p* = 0.023) and weakly associated with concern (Beta = −0.25; *p* = 0.040). We found a higher percentage of variance explained by sociodemographic and clinical factors, ranging from 16% to 28% for the total B-IPQ score and other specific aspects of the illness, such as the personal control of the child, identity, coherence, and emotional representation. Specifically, male parenthood was moderately associated with lower perceptions of emotional representation (Beta = −0.38; *p* = 0.001) and identity (Beta = −0.29; *p* = 0.010) and was weakly associated with lower total B-IPQ scores (Beta = −0.23; *p* = 0.040). Furthermore, age between 7 and 12 years was moderately associated with lower perceptions of child personal control (Beta = −0.35; *p* = 0.002), coherence (Beta = −0.33; *p* = 0.006), and treatment control (Beta = −0.29; *p* = 0.017). Conversely, higher parental education was moderately associated with a higher perception of child personal control (Beta = 0.26; *p* = 0.014) and weakly associated with identity (Beta = 0.24; *p* = 0.026). Finally, previous anaphylaxis, as well as the male gender of the child, were moderately associated with a greater perception of illness identity (Beta = 0.28; *p* = 0.010; Beta = 0.29; *p* = 0.012, respectively).

### 3.4. Influence of SDQ and B-IPQ Scores on FAQLQ-PF

Table 4 shows that a significant portion of the variance (21%) in the FAQLQ-PF scores was added to sociodemographic and clinical factors by the SDQ and B-IPQ scores. In particular, this portion of variance increased to 28% when internalizing problems and specific perceptions of the illness, such as duration, emotional representation, and consequences for the child and parent, were considered.

In this final model, only internalizing problems, timeline, and emotional representation showed a significant positive effect on FAQLQ_PF scores. All coefficients were positive, indicating that higher scores on internalizing problems, timeline, and emotional representation were related to a poorer FAQLQ_PF score.

## 4. Discussion

FA can impact the QoL of patients and families and their psychosocial wellbeing to varying degrees. Illness perception can shape how patients and caregivers cope with and adapt to the challenges of the disease. The present study aimed to investigate the QoL and mental health of children with FA and parental perception of the illness, analyzing possible associations.

Parents reported a moderate impact of FA on the QoL of their children. The total mean score (3.09) is in line with previous studies, which have reported values ranging approximately between 2 and 4 worldwide, depending mainly on the age of the children and the severity of the FA (the greater the age and severity, the greater the impact) [24,31,32,33]. In the present study, an association between QoL and the number of FA, but not previous anaphylaxis, was confirmed. It could be that patients experienced a severe allergic reaction when they were very young and did not remember it or were not fully aware of the real risk. The FAQLQ-PF score was found to be strongly correlated with the emotional difficulties subscale and the macrocategory of internalizing problems, which includes a tendency to worry, have fears, be unhappy, or experience physical complaints and relational difficulties such as problems making friends and social isolation. The hypervigilance required to manage FA, combined with the unpredictable nature of allergic reactions, is a constant source of anxiety and stress for the entire family [10]. As a child, the burden of management usually falls on the caregiver. However, children experience the psychosocial impact of having a FA in how it affects their sense of security, social interactions, and activities. Furthermore, children with FA can be further affected by the effects of caregiver anxiety. Thus, young patients with FA are at risk for anxiety, feelings of isolation, and fear of situations, all of which negatively affect their QoL [13,14]. On the other hand, an impaired QoL can further worsen the emotional and relational wellbeing of patients and families, creating a vicious circle. FAQLQ_PF scores were also found to be correlated with the overall perception of illness and, in particular, with the emotional impact, perceived duration (timeline), and expected effects of the illness on the child’s and parent’s lives. One study [34] reported that 80% of children with milk and egg allergies outgrow them, often by adolescence. Tree nut allergies are the least likely to outgrow, with rates as high as 9%. The age at which allergies resolve varies significantly depending on the allergen, with some resolving in early childhood, and others persisting for years, into adulthood, or even throughout life [34]. It is understandable that a strong illness identity, with the fear that FA will be prolonged and insurmountable, and the burden of the limitations it entails on daily life, may be associated with a greater impact on the emotional wellbeing and QoL of patients and their caregivers [20].

The child’s total difficulties were negatively associated with the parents’ belief in control of their child’s illness and the degree of understanding of the FA, while internalizing problems were negatively associated with parental control of the illness and positively associated with the timeline. Although the cross-sectional design does not allow us to establish causality, we can hypothesize that parents who perceive less control might be more distressed and anxious about managing FA, and, as reported in the literature, this attitude of excessive worry and fear may be transferred to their children [10]. Previous studies showed that parents of children with FA reported higher stress, anxiety and depression compared with healthy controls [10,35,36,37], and maternal anxiety and overprotection have been negatively associated with child QoL and psychosocial health [10,37,38]. On the other hand, greater emotional difficulties in the child (e.g., anxiety and fear of reactions) could lead to the perception that the pathology cannot be sufficiently controlled. In our study, we found that sociodemographic factors explained between 16% and 28% of the variance in the total B-IPQ score and other disease-specific aspects. Specifically, parental male gender was associated with a lower total B-IPQ score and lower perception of emotional representation and identity of the illness. Results are in line with previous research highlighting that mothers of children with FA reported generally higher anxiety and greater stress compared to fathers [15,39]. This is often linked to increased involvement in daily allergy-related care, including meal preparation and social activities. The constant vigilance required for managing FA—such as avoiding allergens, preventing cross-contact, carrying emergency medications, being prepared for a reaction, and educating others—can contribute significantly to chronic stress [15,38,40].

Furthermore, the child’s age between 7 and 12 years was associated with a lower perception of personal control in the child and coherence. Around age 8, children with FA experience significant social and emotional development, often characterized by increased anxiety about accidental exposure due to increased cognitive awareness and independence. This age is also a critical period for managing social situations, such as parties, and transitioning from primary parental safety nets to greater personal responsibility, which requires special attention to building self-confidence and autonomy [10,41]. Qualitative studies on severe FA have shown that parents reported a need to monitor their children’s daily lives and described a feeling of being constantly on high alert. Parents were aware that as their children grew older, they would have to learn to let go, but expanding their scope of action was associated with worry and anxiety [10,42].

Conversely, higher parental education was associated with greater perceptions of the child’s personal control and identity. Parents with a higher level of education tend to have greater knowledge about FA. This knowledge can lead to greater confidence and effectiveness in managing their child’s condition and supporting their needs. On the other hand, a higher level of education can lead to greater awareness of the disease [43].

Finally, previous anaphylaxis was associated with a higher perception of FA identity. Illness identity is a core component of illness perception, representing how a person labels and understands their condition based on their subjective experience of symptoms. This identity involves integrating symptoms into their overall understanding of their health [44,45]. A history of anaphylaxis can lead to an increased illness identity, often linked to the long-term psychological impact, including trauma, worry, and the need for constant vigilance. After anaphylaxis, parents may see FA as a defining, pervasive threat. This can be associated with increased anxiety and a greater perceived risk of future reactions and fatality.

The analysis of the influence of mental health of children and parental perception of FA on QoL, controlling for sociodemographic and clinical factors, showed that a significant portion of the variance in the child QoL was explained by internalizing problems and some specific perceptions of disease. In this final model, higher scores on internalizing problems, timeline, and emotional representation were associated with poorer QoL for the child.

In conclusion, FA can negatively impact the QoL of children and parents through issues with disease perception, such as the feeling of constant risk of exposure and the belief that the allergy is a long-term, insurmountable condition that is difficult to manage. Added to this is a constant monitoring and need for control, which can lead to problems with the emotional representation of the disease, such as increased anxiety, worry, and fear, and a greater risk of social isolation. These parental factors can be passed on to the child. Together, these variables can create a significant burden that can affect the wellbeing of the entire family. The study highlights how parental perceptions are intertwined with the child’s emotions and behaviours, and in some cases, are associated with an impaired QoL. The findings underscore the importance of understanding and exploring the illness perception of caregivers of pediatric patients with FA, as well as paying particular attention to the psychosocial–emotional aspects of the illness in both children and parents. Clinicians should investigate caregivers’ illness perceptions and offer evidence-based information, discussion of doubts and concerns, training in FA and anaphylaxis management, and, when appropriate, referral for psychological support. The B-IPQ can be easily administered at routine allergy appointments to understand and monitor the impact of FA on patients and caregivers after diagnosis and throughout the course of the disease. Tailored interventions aimed at reducing illness identity and emotional representations, enhancing a sense of control, and the use of functional coping strategies are recommended. Group health education, individual counselling, and specific psychological support can be useful tools based on the needs of FA families. A multidisciplinary approach that addresses the medical and psychological aspects of FA should be implemented to ensure adequate QoL.

### Limitations

The study has some limitations that need to be acknowledged. A relatively small sample may limit external validity and generalization by failing to capture the complexity and variability of the population, as the results apply only to the specific and limited group studied. It is easier to introduce bias through non-representative selection methods, which can occur due to convenience or logistical constraints. There is a higher degree of random fluctuation, making it difficult to distinguish real patterns from random variations, and this inherent variability can lead to results that are not representative of the population as a whole. Moreover, the sample consists mainly of patients with severe FA treated at a tertiary-care centre. These sample characteristics may limit the generalizability of the findings. The study also used validated questionnaires completed by parents, which may not fully reflect the patient experience. However, this allowed us to explore the caregiver’s perspective, which could still have an impact on the child’s life. Other factors not considered in this research (e.g., type of allergen, time elapsed since the last anaphylaxis, coping strategies, and parent mental health) could play a role in the wellbeing dynamics of families with FA and should be considered in further research.

## Figures and Tables

**Table 1:** Sociodemographic characteristics and clinical variables of pediatric patients and their parents.

Variables		Total (*n* = 79)	Variables		Total (*n* = 79)
		*n* (%) or Mean (SD)			*n* (%) or Mean (SD)
Children’s characteristics			Clinical variables		
Sex			Previous anaphylaxis		
	Boy	44 (56%)		No	11 (14%)
	Girl	35 (44%)		Yes	68 (86%)
Age (in years)			Number of allergens		
	3–6	23 (29%)		1	13 (16%)
	7–12	56 (71%)		2	12 (15%)
Age (continuous)		7.97 (2.85)		3	17 (22%)
Parents’ characteristics				4	8 (10%)
Sex				5	13 (16%)
	Female	42 (53%)		6	2 (2%)
	Male	37 (47%)		7	14 (18%)
Age (in years)		42.94 (5.75)	Number of allergens (continuous)		3.73 (2.04)
Education level					
	Secondary school or lower	54 (68%)			
	Degree	25 (32%)			

Note. SD = Standard Deviation.

**Table 2:** Descriptive statistics of quality of life and mental health of the pediatric patients, parental perception of the disease, and correlations between measures.

Measures		1	2	3	4	5	6	7	8	9	10	11	12	13	14	15
1	QoL-TOT	--														
2	SDQ-TOT	0.28 *	--													
3	SDQ-INT	0.33 **	0.82 ***	--												
4	SDQ-EXT	0.12	0.81 ***	0.33 **	--											
5	IPQ-CC	0.33 **	0.09	0.13	0.02	--										
6	IPQ-CP	0.28 *	0.02	0.03	0.00	0.79 ***	--									
7	IPQ-T	0.34 **	0.12	0.24 *	−0.04	0.23 *	0.27 *	--								
8	IPQ-PCC	0.11	−0.21	−0.20	−0.15	0.13	0.13	−0.14	--							
9	IPQ-PCP	0.16	−0.27 *	−0.23 *	−0.21	0.06	−0.01	−0.19	0.70 ***	--						
10	IPQ-TC	0.00	−0.11	−0.11	−0.06	0.07	−0.14	−0.23 *	0.34 **	0.32 **	--					
11	IPQ-I	0.08	0.20	0.13	0.20	0.02	0.05	0.35 **	0.03	−0.02	−0.17	--				
12	IPQ-CONC	0.18	0.03	0.02	0.03	0.44 ***	0.52 ***	0.40 ***	−0.10	−0.15	−0.22	0.22	--			
13	IPQ-COHE	−0.02	−0.24 *	−0.22	−0.17	−0.02	−0.08	−0.29 *	0.43 ***	0.45 ***	0.38 ***	−0.29 *	−0.12	--		
14	IPQ-E	0.40 ***	0.13	0.09	0.13	0.37 ***	0.51 ***	0.28 *	−0.01	−0.12	−0.26 *	0.35 **	0.60 ***	−0.06	--	
15	IPQ-TOT	0.42 ***	0.00	0.01	−0.01	0.64 ***	0.66 ***	0.43 ***	0.46 ***	0.35 **	0.19	0.42 ***	0.59 ***	0.21	0.63 ***	--
Mean (SD)		3.09 (1.17)	10.99 (5.88)	5.47 (3.62)	5.52 (3.58)	7.10 1.95	6.59 2.26	6.91 2.40	2.65 2.03	2.08 1.72	1.57 2.16	6.54 2.75	7.53 2.07	1.68 1.74	6.61 2.61	49.22 10.20
Median (IQ)		2.98 (1.82)	11.00 (10.00)	5.00 (5.00)	5.00 (4.00)	8.00 (2.00)	7.00 (3.00)	7.00 (4.00)	2.00 (2.00)	2.00 (2.00)	1.00 (2.00)	7.00 (4.00)	8.00 (2.00)	1.00 (1.00)	7.00 (3.00)	51.00 (14.00)

Note: TOT = Total score; SDQ subscales: INT = Internalizing problems, EXT = Externalizing problems; B-IPQ subscales: COC = Consequences child, COP = Consequences parent, T = Timeline, PCC = Personal control child, PCP = Personal control parent, TC = Treatment control, I = Identity, CONC = Concern, COHE = Coherence, E = Emotions; * *p* < 0.05; ** *p* < 0.01; *** *p* < 0.001.

**Table 3:** Influence of sociodemographic and clinical factors on quality of life and mental health of the pediatric patients, and on parental perception of the disease.

Measures	Sex Patient (Boy) B (SE)	Age Patient (7–12 Years) B (SE)	Number of Allergens B (SE)	Previous Anaphylaxis (Yes) B (SE)	Sex Parent (Male) B (SE)	Age Parent B (SE)	Educational Level Parent (Degree) B (SE)	R-Square
QoL-TOT	−0.45 (0.28)	−0.06 (0.31)	0.15 (0.07) *	−0.08 (0.39)	−0.45 (0.27)	0.02 (0.02)	−0.21 (0.29)	0.13
SDQ-INT	−0.56 (0.90)	1.19 (0.98)	0.02 (0.21)	−0.25 (1.24)	−0.29 (0.86)	−0.12 (0.08)	−0.08 (0.92)	0.07
SDQ-EXT	0.84 (0.89)	0.51 (0.97)	0.15 (0.21)	−0.12 (1.23)	−1.17 (0.85)	0.09 (0.08)	−0.55 (0.91)	0.07
SDQ-TOT	0.28 (1.49)	1.71 (1.62)	0.17 (0.35)	−0.37 (2.06)	−1.46 (1.43)	−0.03 (0.13)	−0.63 (1.52)	0.04
B-IPQ-CC	−0.36 (0.48)	−0.39 (0.52)	0.17 (0.11)	0.05 (0.67)	−0.24 (0.46)	−0.04 (0.04)	−0.03 (0.49)	0.08
B-IPQ-CP	0.19 (0.54)	−0.16 (0.59)	0.21 (0.13)	0.80 (0.75)	−1.20 (0.52) *	0.03 (0.05)	0.14 (0.55)	0.14
B-IPQ-T	1.10 (0.59)	0.82 (0.64)	0.07 (0.14)	−0.13 (0.82)	−0.58 (0.57)	−0.02 (0.05)	0.64 (0.60)	0.09
B-IPQ-PCC	−0.60 (0.45)	−1.55 (0.48) **	0.26 (0.10) *	−0.43 (062)	0.40 (0.43)	−0.03 (0.04)	1.15 (0.45) *	0.28 **
B-IPQ-PCP	−0.56 (0.43)	−0.79 (0.46)	0.05 (0.10)	−0.12 (0.59)	0.33 (0.41)	−0.01 (0.04)	0.46 (0.43)	0.08
B-IPQ-TC	−0.36 (0.51)	−1.35 (0.55) *	0.17 (0.12)	−1.17 (0.70)	0.66 (0.49)	−0.03 (0.04)	0.81 (0.52)	0.17
B-IPQ-I	1.57 (0.61) *	1.08 (0.66)	−0.06 (0.15)	2.21 (0.83) *	−1.56 (0.59) *	−0.02 (0.05)	1.40 (0.62) *	0.27 **
B-IPQ-CONC	0.44 (0.51)	0.55 (0.55)	0.02 (0.12)	0.35 (0.70)	−1.02 (0.49) *	−0.01 (0.04)	−0.53 (0.52)	0.09
B-IPQ-COHE	−0.60 (0.41)	−1.25 (0.45) **	−0.05 (0.10)	−0.11 (0.57)	0.71 (0.39)	−0.02 (0.04)	0.34 (0.42)	0.16
B-IPQ-E	0.58 (0.61)	0.56 (0.66)	0.13 (0.14)	0.25 (0.84)	−1.97 (0.58) **	−0.00 (0.05)	−0.50 (0.62)	0.18 *
B-IPQ-TOT	1.51 (2.38)	−2.84 (2.58)	0.94 (0.56)	1.43 (3.28)	−4.76 (2.28) *	−0.12 (0.21)	4.11 (2.42)	0.18 *

Note: TOT = Total score; SDQ subscales: INT = Internalizing problems, EXT = Externalizing problems; B-IPQ subscales: CC = Consequences child, CP = Consequences parent, T = Timeline, PCC = Personal control child, PCP = Personal control parent, TC = Treatment control, I = Identity, CONC = Concern, COHE = Coherence, E = Emotions; B = Unstandardized regression coefficient; SE = Standard Error; * *p* < 0.05; ** *p* < 0.01.

**Table 4:** Influence of mental health of children and parental perception of the disease on quality of life of the pediatric patients.

Predictors	B (SE)	Beta	Delta R-Square
Model 1			0.21 ***
SDQ-TOT	0.05 (0.02) *	0.26	
B-IPQ-TOT	0.05 (0.01) ***	0.43	
Model 2			0.28 ***
SDQ-INT	0.08 (0.03) *	0.24	
B-IPQ-CC	0.14 (0.10)	0.23	
B-IPQ-CP	−0.10 (0.10)	−0.19	
B-IPQ-T	0.11 (0.05) *	0.23	
B-IPQ-E	0.14 (0.05) *	0.31	

Note: TOT = Total score; SDQ-INT = Internalizing problems; B-IPQ subscales: CC = Consequences child, CP = Consequences Parent, T = Timeline, E = Emotions; B = Unstandardized regression coefficient; SE = Standard Error; Beta = Standardized regression coefficient; * *p* < 0.05; *** *p* < 0.001.

## Data Availability

Further data are available from the corresponding author upon reasonable request. The data are not publicly available due to privacy.
